# Cooccurrence of prey species alters the impact of predators on prey performance through multiple mechanisms

**DOI:** 10.1002/ece3.4413

**Published:** 2018-08-11

**Authors:** Clifton B. Ruehl, Heather Vance‐Chalcraft, David R. Chalcraft

**Affiliations:** ^1^ Department of Biology East Carolina University Greenville North Carolina

**Keywords:** competition, geometric morphometrics, nonconsumptive effects, nonlethal effects, per capita food availability, phenotypic plasticity

## Abstract

When prey are differentially affected by intra and interspecific competition, the cooccurrence of multiple prey species alters the per capita availability of food for a particular prey species which could alter how prey respond to the threat of predation, and hence the overall‐effect of predators. We conducted an experiment to examine the extent to which the nonconsumptive and overall effect of predatory water bugs on snail and tadpole traits (performance and morphology) depended on whether tadpoles and snails cooccurred. Tadpoles and snails differed in their relative susceptibility to intraspecific and interspecific competition, and predators affected both prey species via consumptive and nonconsumptive mechanisms. Furthermore, the overall effect of predators often depended on whether another prey species was present. The reasoning for why the overall effect of predators depended on whether prey species cooccurred, however, differed for each of the response variables. Predators affected snail body growth via nonconsumptive mechanisms, but the change in the overall effect of predators on snail body growth was attributable to how snails responded to competition in the absence of predators, rather than a change in how snails responded to the threat of predation. Predators did not affect tadpole body growth via nonconsumptive mechanisms, but the greater vulnerability of competitively superior prey (snails) to predators increased the strength of consumptive mechanisms (and hence the overall effect) through which predators affected tadpole growth. Predators affected tadpole morphology via nonconsumptive mechanisms, but the greater propensity for predators to kill competitively superior prey (snails) enhanced the ability of tadpoles to alter their morphology in response to the threat of predation by creating an environment where tadpoles had a higher per capita supply of food available to invest in the development of morphological defenses. Our work indicates that the mechanisms through which predators affect prey depends on the other members of the community.

## 1 INTRODUCTION

Predators can affect prey populations by killing prey (i.e., a consumptive effect or CE) or by scaring them (i.e., a nonconsumptive effect or NCE). Consumption of prey alters prey abundance (Dorn, 2013; Sih, Crowley, Mcpeek, Petranka, & Strohmeier, 1985; Turner & Chislock, 2007), influences the growth of survivors (Van Buskirk & Yurewicz, 1998), and can produce density‐mediated trophic cascades that alter the availability of food resources for prey (Hairston, Smith, & Slobodkin, 1960; Oksanen, Fretwell, Arruda, & Niemela, 1981). NCEs of predators include changes in prey phenotype that may reduce the likelihood of prey being killed by the predator (Tollrian & Harvell, 1999) and cause trait‐mediated trophic cascades (Preisser, Bolnick, & Benard, 2005; Ruehl & Trexler, 2013; Werner & Peacor, 2003). A number of studies have revealed that NCEs of predators can account for a large fraction of the overall effect of predators (Peacor, Pangle, Schiesari, & Werner, 2012; Peacor & Werner, 2001; Ruehl & Trexler, 2013; Werner & Peacor, 2006) but some have found that the NCE of predators contributes little to the overall effect relative to the contribution of the CE of predators (e.g., Hoverman & Relyea, 2012).

The environment where predator‐prey interactions occur could impact the strength of NCEs as prey must balance inducing behaviors, morphologies or life history strategies that reduce their risk of predation with increased odds of reduced growth, lower fecundity, or starvation from competition (Peckarsky et al., 2008; Preisser et al., 2005; Schmitz & Trussell, 2016; Werner & Peacor, 2003). For example, theoretical (Luttbeg, Rowe, & Mangel, 2003; Peacor & Werner, 2004; Turner, 2004) and empirical work (Bolnick & Preisser, 2005; Davenport & Chalcraft, 2014; McCoy, 2007) has found that per capita food availability for prey alters NCEs of predators on prey.

A meta‐analysis revealed that the impact of food availability on NCEs depended on whether per capita food availability was altered by a change in the number of competitors of prey present or by a direct change in the density of food available for prey (Bolnick & Preisser, 2005). Changes in the number of individuals that compete with prey had stronger effects on the NCEs of predators on prey than did manipulations of food availability for prey. The meta‐analysis of Bolnick and Preisser (2005) also revealed that the NCE on responses related to prey growth and development rate were stronger when fewer competitors were present as expected by theory, but the NCE on responses related to mortality risk was stronger when more competitors were initially present. The stronger NCE of predators on responses related to mortality risk when more competitors were present may result from the elevated stress of predation risk in conjunction with a low amount of food available for prey. Conversely, prey with lots of food available and few competitors are less likely to die from predator induced stress.

Most theoretical models have not explicitly incorporated the influence of interspecific competition on the NCE of predators. Peacor and Werner (2004) developed a theoretical model that predicts the NCE of a predator on prey growth when prey have interspecific competitors, but their model assumes that the NCE of predators on the per capita foraging rate of prey is not influenced by either intraspecific or interspecific competition (i.e., the 1‐Δ in their model does not depend on the influence of intraspecific or interspecific competition). Rather, the Peacor and Werner model presumes that prey will always change their behavior in the same way when predators are present regardless of how much food is available. This presumption may not always be true as prey may be more likely to engage in risky behaviors when there is a big foraging benefit (Abrams, 1991; Brown & Kotler, 2004; Gilliam & Fraser, 1987) or decrease risky foraging because energy needs are being met (Luttbeg et al., 2003). Furthermore, reductions in prey foraging behavior in response to predators could diminish after prey develop morphological defenses that reduce their risk of predation (Relyea, 2003). The ability to develop morphological defenses may require the acquisition of sufficient food resources to invest in the development of energetically costly morphological defenses (Ruehl & Trexler, 2013). Though other models (Abrams, 1992; Peacor & Werner, 2004; Turner, 2004) do not explicitly incorporate the influence of interspecific competitors, they do suggest that the NCE of a predator will change as per capita food availability for prey changes.

If the NCE of predators on prey depends on per capita food availability for prey, variation in competitive ability to deplete food resources among prey species should cause the NCE of predators on a focal prey species to depend on the cooccurrence of the focal prey species with other prey species. For example, a competitively superior prey species would deplete food faster, leaving less for the focal prey species compared to when the focal prey species occurred alone (i.e., interspecific competition > intraspecific competition). This cooccurrence with a competitively superior prey species should weaken NCEs of predators on the growth and development of focal prey but enhance the NCE of predators on mortality risk of focal prey when multiple prey species cooccur. In contrast, a competitively inferior prey species would deplete food more slowly than a focal prey species (i.e., intraspecific competition > interspecific competition) relative to when the focal prey species occurred alone. This situation should strengthen NCEs of predators on the growth and development of focal prey but weaken the NCE of predators on prey mortality risk when multiple prey species cooccur.

Some have suggested that the NCE could explain much of the overall effect (combined consumptive and NCEs) of predators (Bolnick & Preisser, 2005; Peacor & Werner, 2004; Peckarsky et al., 2008; Werner & Peacor, 2003). As a result, we expect that any changes to the NCE of predators due to the cooccurrence of multiple prey will also alter the overall effect of predators on their prey. Though others (Peacor & Werner, 2000; Relyea, 2000; Werner, 1991) have assessed the NCE of predators on prey in the presence of interspecific competitors, they have not explicitly evaluated whether differences in the susceptibility of prey to interspecific and intraspecific competition influences the NCE of predators on prey growth, development or mortality risk. For example, prior field studies (1) have not directly assessed how either growth or mortality risk of prey species were affected by intraspecific and interspecific competition, (2) were too short in duration to report whether NCE on prey growth, mortality or development changed as the ratio of intraspecific to interspecific competitors changed (Werner, 1991), (3) always exposed focal prey species to a fixed density of interspecific competitors that prevented an evaluation of how focal prey would perform in the absence of interspecific competition (e.g., Peacor & Werner, 2000), and (4) only exposed prey to predators when multiple prey species cooccurred and did not assess prey performance in response to predators when only a single prey species was present (e.g., Relyea, 2000). Werner (1991) did find, however, that caged predators differentially affected the growth of two cooccurring prey species and that each prey species reduced their foraging activity less when both prey species cooccurred than when they occurred alone. Werner's (1991) results suggests that predators would have exerted stronger NCE on the growth, development and mortality risk of each prey species (assuming foraging activity is related to prey growth, development, and mortality risk) when they did not cooccur with each other, but this hypothesis was not assessed.

In this study, we used a tri‐trophic food chain to examine the extent to which differences in the relative susceptibility to intraspecific and interspecific competition between functionally similar, but phylogenetically distant, prey species that competed for a shared food resource influenced the nonconsumptive and overall effects of a shared predator on each prey species. We expected that both the NCE and overall effects of predators on prey would be stronger when prey species were more vulnerable to interspecific, relative to intraspecific, competition, while both the NCE and overall effects would weaken when prey species were less vulnerable to interspecific, relative to intraspecific, competition. We predicted that the NCE and overall effects would cascade to benefit algal abundance and this effect would be larger for the more vulnerable prey species.

## 2 METHODS

### 2.1 Study system

We examined the extent to which the overall effect and NCE of predatory giant water bugs (*Belastoma flumenium* Say) on two prey species change when multiple prey species cooccur relative to when a single prey species is present with the predator. The prey species we examined were pulmonate snails (*Physa acuta*, Draparnaud) and tadpoles of Cope's grey treefrog (*Hyla chrysoscelis*, Cope*)*. All three species often cooccur in temporary ponds. Pulmonate snails and tadpoles both compete for common food resources (periphyton and detritus) and are prey to water bugs. Water bugs are known to have important NCEs and CEs on multiple snail (Hoverman & Relyea, 2012; Kesler & Munns, 1989; Turner & Chislock, 2007) and tadpole species (Babbitt & Jordan, 1996; Swart & Taylor, 2004). However, little work has been done on the interactions between *P. acuta, H. chrysoscelis*, and *B. flumenium*.

### 2.2 Experimental design

Our experiment consisted of three predator treatments applied to each of three grazing treatments which differed in the relative abundance of the two‐prey species (producing nine treatments) plus a tenth treatment that contained no predators or prey to determine algal levels in the absence of predators and prey. The three predator treatments were (1) the presence of free‐swimming (i.e., uncaged) water bugs, (2) the presence of caged water bugs fed prey to transmit the threat of predation via waterborne chemical cues to prey outside of the cage (Chalcraft, Binckley, & Resetarits, 2005; Hoverman & Relyea, 2012; Ruehl & Trexler, 2013), and (3) no water bugs present. The three grazing treatments were represented by (1) 100 snails present, (2) 100 tadpoles present, and (3) 50 snails and 50 tadpoles present. The substitutive design associated with our three grazing treatments allows us to evaluate the relative influence of interspecific and intraspecific competition. Each of the 10 treatments was replicated once within each of five spatial blocks of artificial ponds (i.e., mesocosms).

We used cattle watering tanks for mesocosms. Four blocks (40 mesocosms) contained 189‐L tanks, while the fifth block (10 mesocosms) contained 378‐L tanks. Limited availability prevented the use of mesocosms that were the same size, but the two sizes only varied in height; their surface area was the same (approximately 1.13‐m^2^) and all mesocosms were filled with water to the same depth (approx. 25 cm). Placing mesocosms of different sizes into different spatial blocks allowed us to account for any effects of mesocosm size in the block effect of statistical models.

Mesocosms were equipped with a standpipe to regulate water depth and covered with shade cloth to prevent colonization or escape of animals. We added 300‐g of mixed hardwood leaf litter and a 1‐L inoculum of pond water containing phytoplankton, zooplankton, and periphyton to stimulate primary productivity on 18 June 2010. All tanks received a floating water bug cage (opaque plastic cup perforated with pin sized holes and covered with window screen) that allowed water exchange in predator‐cue treatments and a periphytometer (white flagging tape) attached to the tank wall to facilitate the estimation of algal biomass in mesocosms.

Treatments were randomly assigned to one mesocosm within each block and newly hatched larvae of *H. chrysoscelis* (mean mass ± 1 *SE*, 5.85 ± 0.11 mg) and newly produced offspring of *P. acuta* (mean mass ± 1 *SE*, 4.77 ± 0.11 mg) were counted and the appropriate number of each species (depending on treatment) were assigned to each mesocosm on 23 June 2010 (see Supporting Information Appendix S1 for details). We collected water bugs (mean mass ± 1 *SE* = 0.167 ± 0.009 g) from a fishless pond and placed one *Belostoma* into each mesocosm designated to contain water bugs or released one *Belostoma* into the mesocosm to roam freely in the uncaged treatments on 24 June 2010. Caged water bugs were fed four individual prey items every 4 days. The ratio of prey species fed to predators corresponded to the initial relative abundance of prey species present in the mesocosm. For example, four tadpoles were fed to water bugs in the treatment with tadpoles but no snails, while two snails and two tadpoles were fed to predators in the treatment with both tadpoles and snails. One water bug died on 4 July and was replaced the same day with a similar sized individual.

We began to destructively sample mesocosms 30 days after predators were added as tadpoles began to metamorphose (front limb eruption, Gosner, 1960). One block was sampled on each of 5 days (19 July–23 July). Surviving snails and tadpoles were collected by draining the water from mesocosms through a net and searching mesocosm walls and the litter for any adult snails or tadpoles that remained. We did not collect snails born in the tanks during the experiment for logistical reasons given their large numbers and incredibly small body size. Periphytometers and snails from each mesocosm were frozen for later processing. Tadpoles were processed alive in the lab before releasing them to their parental pond. We processed tadpoles by counting survivors, measuring their mass, and taking pictures of the lateral view of 16 haphazardly chosen individuals from each mesocosm to facilitate quantifying tadpole shape variation. Snails were thawed and soft tissue was separated from the shell with forceps before recording the wet snail tissue mass. We haphazardly chose 16 snails from each mesocosm to photograph for shape analysis. Snails were oriented aperture side down with the shell apex pointed away from the researcher prior to capturing images.

### 2.3 Response variables

We estimated instantaneous mortality rates (*m*) of snails and tadpoles for each mesocosm as *m* = −log (#alive in the mesocosm at end of experiment/#present in the mesocosm at the beginning of the experiment) (see Supporting Information Appendix S1 for details) (e.g., Billick & Case, 1994). The amount of soft body growth (change in mass of individuals) of each species was calculated for each mesocosm (see Supporting Information Appendix S1 for details). We also quantified snail and tadpole body shape using geometric morphometrics (see Supporting Information Appendix S1 for details). We did not characterize shape for snails from consumptive treatments because there were too few individuals to adequately measure shape. The geometric morphometric analysis resulted in the production of six shape variables that described 90% of the overall variation in tadpole shape and five shape variables that described 91% of the overall variation in snail shape. Given that we had multiple observations for body growth and body shape within each mesocosm, we obtained the average values for body growth and body shape within each mesocosm prior to further statistical analysis to avoid pseudoreplication. Algal abundance was estimated in each mesocosm with a spectrophotometer by extracting chlorophyll from periphyton scraped from a known surface area of the periphytometer in the mesocosms (see Supporting Information Appendix S1 for details).

### 2.4 Analyses

For each prey species, we used general linear mixed models to analyze growth and survivorship data and MANCOVA for body shape data. Given that these analyses focused on the response of individual species, each analysis only included data from those treatments in which the species was present. Our mixed models specified treatments as a fixed factor but mixed models also specified block as a random factor. A generalized linear mixed model with a binomial error distribution and logit function was performed on survival data (proportion surviving as dependent variable) and produced qualitatively similar results to the general linear mixed model on our estimates of instantaneous mortality rates. We present the results from the general linear model for ease of interpretation as the instantaneous death rate (log transformed survival data) has direct biological meaning while estimates from a generalized linear model with a logit link function would require back transformation to yield a response variable (proportion surviving) that is more easily interpretable. The MANCOVA specified the effects of treatment, block and a measure of body size (i.e., centroid size obtained from the Procrustes superimposition conducted in the process of deriving shape variables; a multivariate measure of body size) to account for variation in shape due to body size (i.e., multivariate allometry). The slope of the relationship between shape and body size was consistent among treatments for both tadpoles and snails so we did not include an interaction between size and treatment for the final analyses on body shape. Multivariate effect sizes were calculated using Wilks’ partial‐eta squared ($\eta_{p}^{2}$; Tabachnick & Fidell, 2007).

We assessed several hypotheses about the effects of competitors and predators on the growth, survivorship, and body shape of each prey species using planned contrasts in conjunction with our general linear mixed models and MANCOVA (Supporting Information Appendix S2). The first hypothesis is that each prey species is equally susceptible to interspecific and intraspecific competition (Contrast 1; Supporting Information Appendix S2). Hypotheses about the effects of predators include (i) the cooccurrence of multiple prey species does not alter either the NCE (Contrast 2) or overall effect (Contrast 3) of predators on a particular prey species, (ii) the magnitude of the NCE (Contrasts 4a–c) and overall (Contrasts 5a–c) effect of predators on each prey species is zero, (iii) the degree to which the NCE and overall effect of predators on a prey species differed from each other did not depend on whether multiple prey species cooccurred (Contrast 6) and (iv) the NCE of a predator on a prey species was equivalent to the overall effect of predators (Contrasts 7a–c). We assessed hypotheses about whether the magnitude of the NCE, the overall effect of predators or the degree of equivalence in the NCE and overall effect differed from zero under each of three contexts (i) regardless of the cooccurrence of multiple prey species (Contrast/hypotheses 4a, 5a, and 7a), (ii) when the focal prey species is the only prey species present (Contrast/hypotheses 4b, 5b, and 7b) and (iii) when the focal prey species cooccurs with another prey species (Contrast/hypotheses 4c, 5c, and 7c). Depictions of shape change for significant planned contrasts were generated using tpsREGR (Rohlf, 2011) software that used landmarks to create a consensus configuration.

Free‐swimming predators could cause the average shape of prey in mesocosms to differ from that observed in mesocosms where there were no predators or when there were caged predators because predators were unable to capture and kill individuals on one end of the shape distribution (or preferred to eat individuals on one end of the shape distribution) that would be observed in the absence of predators (i.e., a CE). If free‐swimming predators were unable to capture and kill individuals on one end of the shape distribution, the distribution of shape scores within mesocosms having free‐swimming predators would be a truncated version of the distribution of shape scores in mecocosms where predators could not kill prey. We evaluated whether the shape of the distribution of standardized prey shape scores within mesocosms in one treatment differed from that observed within mesocosms of a second treatment using two sample Kolmogorov–Smirnov (KS) tests. Shape scores within mesocosms were standardized in order to remove variation in shape scores of individuals among mesocosms within a treatment so that we could test for differences in the distribution of shape scores within mesocosms between treatments. A separate KS test was completed for each pair of predator treatments and for each of the shape variables. A similar approach was implemented to compare distributions of prey body growth when we found evidence to suggest that predators altered the average body growth of a prey species as predators may have preferred to consume faster growing prey.

We were also interested in evaluating hypotheses about how competitors and predators affected the abundance of chlorophyll *a* present. To do this, we performed planned contrasts (Supporting Information Appendix S3) in conjunction with a mixed linear model that specified chlorophyll *a* abundance as the dependent variable, treatment as a fixed effect and block as a random effect. Given that all treatments had algae in them, data from all treatments were included in this analysis. These hypotheses are that (i) prey species had similar abilities to suppress algal abundance (Contrast 8), (ii) the presence of a single prey species did not suppress algal abundance (Contrast 9a–c), and (iii) the presence of two prey species had a similar effect in reducing algal abundance as the presence of one prey species (Contrast 10a–c). The hypothesis that a single prey species does not affect algal abundance was assessed when (a) either, but not both, prey species was present (Contrast 9a), (b) snails were the only prey present (Contrast 9b) and (c) tadpoles were the only prey present (Contrast 9c). We tested the hypothesis that two prey species had a similar effect to that observed when one prey species was present by comparing the response with two prey species to treatments with (a) either, but not both prey species present (Contrast 10a), (b) snails were the only prey present (Contrast 10b) and (c) tadpoles were the only prey present (Contrast 10c).

We also evaluated hypotheses pertaining to how the overall and NCEs of predators impacted algal abundance. The hypotheses pertaining to the indirect effects of predators on algal abundance included (i) predators exerted a similar NCE (Contrast 11) and overall effect (Contrast 12) on algae in environments that only contained snail prey as observed in environments that only contained tadpole prey, (ii) predators do not influence algal abundance via either a NCE (Contrast 13a–c) or overall effect (Contrast 14a–c) when one prey species was present, (iii) predators exerted a similar NCE (Contrast 15a–c) or overall effect (Contrast 16a–c) on algal abundance when one or two prey species were present, and (iv) the NCE of predators on algal abundance was similar to the overall effect of predators on algal abundance (Contrast 17a–d). We include multiple hypotheses for some contrasts (contrast numbers that also have lower case letters associated with them) because the contrasts evaluate the same hypothesis in different contexts. For example, the hypotheses that the NCE and overall effect of predators on algal abundance are not different from each other (Contrast 17) when (a) snails are the only prey present, (b) tadpoles are the only prey present, (c) both snails and tadpoles are present, and (d) the difference in the NCE and overall effect of predators on algae does not depend on whether 1 or 2 prey species are present.

We report estimates of effects (e.g., the degree to which the effect of a predator depends on context or the degree to which the effect of a predator differs from zero) as percent changes in the value of the response variable and probability values associated with null hypotheses from our planned contrasts. We present some nonzero effect estimates that could be considered not statistically different from zero (i.e., *p* > 0.05) because the estimate may statistically differ from another estimate that is also not statistically different from zero (e.g., NCE of predators on snails in the presence of tadpoles). In such a case, it is illogical to conclude that both estimates have a value of zero (i.e., there is no effect) and differ from each other at the same time. The approach we take is consistent with the recommendations made by the American Statistical Association (Wasserstein & Lazar, 2016) and others (Hurlbert & Lombardi, 2009, 2012) for the proper use and interpretation of *p*‐values. Given many of the issues associated with attempts to control experimentwise error rate, we report unadjusted *p*‐values for all contrasts as suggested by others (Althouse, 2016; Feise, 2002; Hurlbert & Lombardi, 2009, 2012; Rothman, 1990; Rubin, 2017). We used SAS version 9.4 for windows to complete all analyses (SAS Institute Inc., Cary, NC, USA).

## 3 RESULTS

### 3.1 Effects of competition (in the absence of predators)

**Figure 1:**
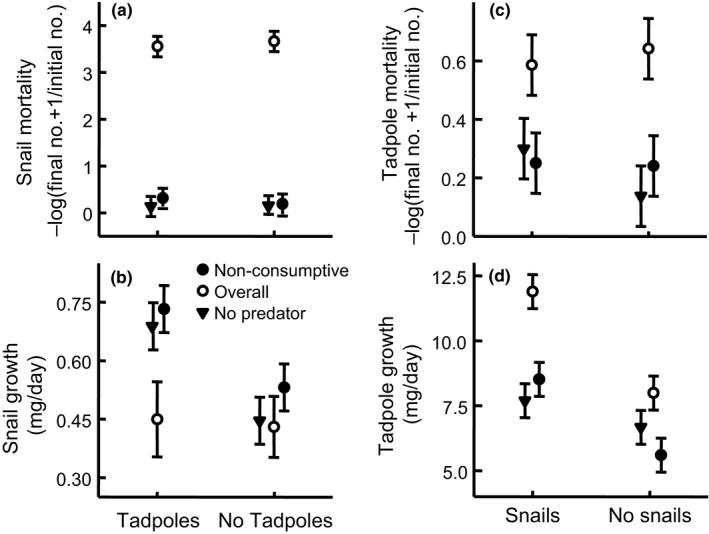
Mortality and growth (mean ± *SE*) for snails (a–b) and tadpoles (c–d) in response to nonconsumptive water bugs (nonconsumptive effect, filled circle), free‐swimming water bugs (overall effect, open circle), and no water bugs (filled triangle). Snails and tadpoles occurred together at half the density of mesocosms with only a single prey species. Note that size differences between tadpoles and snails required that the axes differ between the left and right panel

The replacement of intraspecific competitors with interspecific competitors had little impact on snail mortality (Contrast 1: *t*_20_ = 0.05, *p* = 0.96; Figure 1a) or shell shape (Contrast 1: *F*_5, 11_ = 0.92; *p* = 0.50; $\eta_{p}^{2}$ = 0.30). Replacing intraspecific with interspecific competitors, however, enhanced snail growth by 54% (Contrast 1: *t*_14.8_ = 3.13; *p* = 0.007; Figure 1b). The replacement of intraspecific competitors with interspecific competitors increased tadpole mortality by 118% (Figure 1c) and enhanced tadpole growth by 15% (Figure 1d) but neither of these effects were statistically different from zero (Contrast 1: mortality–*t*_24_ = 1.11, *p* = 0.28; growth–*t*_20_ = 1.30; *p* = 0.21). Furthermore, the presence of snails did not cause tadpoles to alter their shape (Contrast 1: *F*_6, 9_ = 0.35; *p* = 0.89; $\eta_{p}^{2}$ = 0.19).

### 3.2 Effects of predators on snails

The impact of free‐swimming water bugs on snail mortality did not depend on whether prey species cooccurred (Contrast 3: *t*_20_ = 0.21, *p* = 0.83). Overall, free‐swimming water bugs increased snail mortality (Contrast 5a: *t*_20_ = 16.06; *p* < 0.001; overall effect in Figure 1a) by 2.339% relative to when predators were absent. Caged water bugs had little impact on snail mortality (Contrast 4a: *t*_20_ = 0.49, *p* = 0.63; Figure 1a) and the impact did not depend on whether prey species cooccurred or not (Contrast 2: *t*_20_ = 0.32, *p* = 0.75). Consequently, free‐swimming water bugs inflicted greater mortality on snails than caged water bugs (Contrast 7a: *t*_20_ = 15.57, *p* < 0.001) and the difference in the impact of caged and free‐swimming water bugs on mortality did not depend on whether prey species cooccurred or not (Contrast 6: *t*_20_ = −0.54, *p* = 0.60; Fiure 1a).

**Figure 2:**
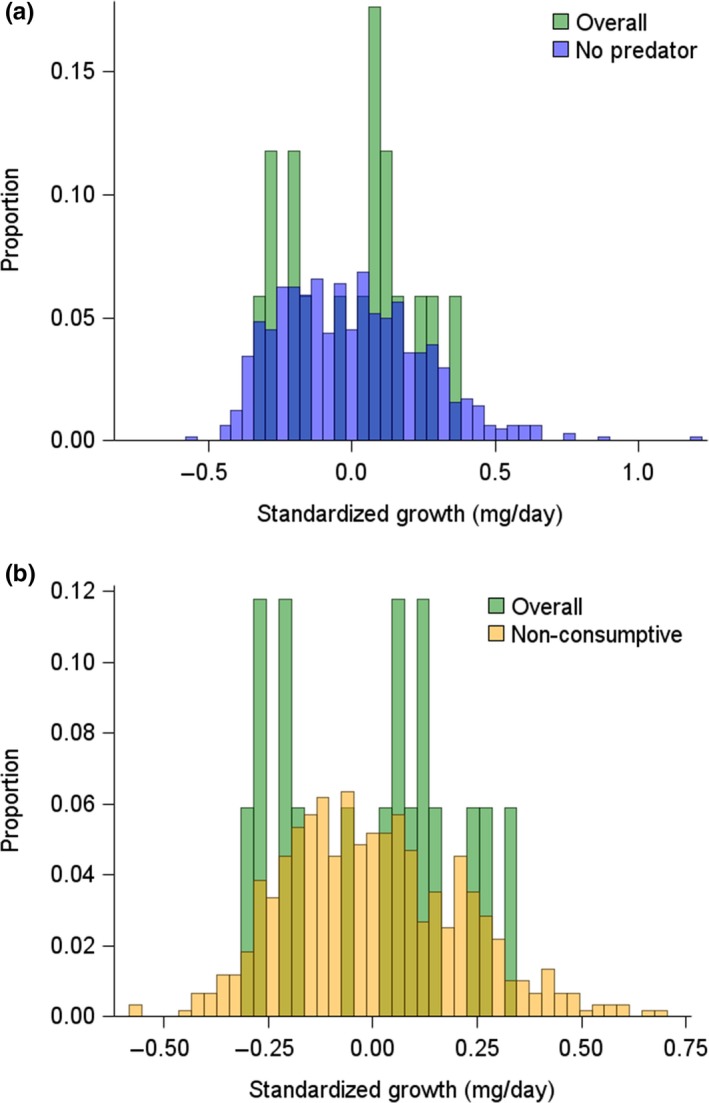
Distribution of standardized snail growth in treatments with a free‐swimming water bug (Overall, green) relative to distributions within treatments that (a) have no water bug present (No Predator, blue) and (b) a caged water bug present (Nonconsumptive, orange). Growth of individuals were standardized by subtracting an individual's growth rate from the average growth rate of all individuals within the same mesocosm to account for differences in the average growth rate among mesocosms within a treatment [Colour figure can be viewed at http://wileyonlinelibrary.com]

When snails occurred with tadpoles, free‐swimming water bugs reduced snail growth by 35% compared to when water bugs were absent (Contrast 5c: *t*_15.78_ = 2.22; *p* = 0.04; Figure 1b). We lack sufficient statistical evidence to reject the hypothesis that free‐swimming water bugs had the same effect on snail growth in the absence of tadpoles (Contrast 3: *t*_15.75_ = 1.57; *p* = 0.14), but free‐swimming water bugs only reduced snail growth by 4% when snails did not occur with tadpoles (Contrast 5b: *t*_15.43_ = 0.17; *p* = 0.87; Figure 1b). Caged water bugs had a negligible, but positive, effect on snail growth (Contrast 4a: *t*_14.85_ = 1.19; *p* = 0.25) and this effect did not depend on whether snails cooccurred with tadpoles (Contrast 2: *t*_14.85_ = 0.38; *p* = 0.71; Figure 1b). Snail growth in the presence of free‐swimming water bugs was 39% slower than in the presence of caged water bugs when snails and tadpoles cooccurred (Contrast 7c: *t*_15.78_ = 2.63; *p* = 0.02). In contrast, when tadpoles were absent, free‐swimming water bugs only caused snails to grow 19% slower than that observed in the presence of caged predators and this reduction in growth was not statistically different from zero (Contrast 7b: *t*_15.43_ = 1.10; *p* = 0.29, Figure 1b). Despite the fact that the degree to which free‐swimming and caged predators differ in their effect on snail growth varies among environments that either contain or lack tadpoles, we lack strong statistical evidence to reject the hypothesis that the extent to which free‐swimming and caged predators differ in their effect on snail growth is the same in environments that contain tadpoles as that observed in environments without tadpoles (Contrast 6: *t*_15.75_ = 1.28; *p* = 0.22; Figure 1b). On average, free‐swimming water bugs caused snails to grow 30% more slowly than did caged water bugs (Contrast 7a: *t*_15.51_ = 2.73; *p* = 0.02). Despite observing that free‐swimming water bugs altered the average body growth of snails, the shape of the distribution of snail body growth in mesocosms with free‐swimming predators was not different from the shape of the distribution of snail body growth in mesocosms that contained either caged water bugs or no water bugs (*p* ≥ 0.622 in both cases, Figure 2). There was no evidence of snail shell shape variation in response to caged water bugs (Contrast 4a: *F*_5, 11_ = 0.167; *p* = 0.66; $\eta_{p}^{2}$ = 0.23).

### 3.3 Effects of predators on tadpoles

The impact of free‐swimming water bugs on tadpole mortality did not depend on whether prey species cooccurred (Contrast 3: *t*_24_ = 1.05; *p* = 0.30). Overall, free‐swimming water bugs increased tadpole mortality by 287% (Contrast 5a: *t*_24_ = 3.82, *p* = 0.001; overall effect in Figure 1c) relative to when predators were absent. Caged water bugs had little impact on tadpole mortality (Contrast 4a: *t*_24_ = 0.26, *p* = 0.80; Figure 1a,c), and the impact did not depend on whether prey species cooccurred or not (Contrast 2: *t*_24_ = 0.74, *p* = 0.47). Consequently, free‐swimming water bugs inflicted greater mortality on tadpoles than caged water bugs (Contrast 7a: *t*_24_ = 3.56, *p* = 0.002) and the difference in the impact of caged and free‐swimming water bugs on mortality did not depend on whether prey species cooccurred or not (Contrast 6: *t*_24_ = 0.32, *p* = 0.75; Figure 1a,c).

Free‐swimming water bugs stimulated tadpole growth by 55% when they cooccurred with snails (Contrast 5c: *t*_20_ = 5.33; *p* < 0.001), but their effect changed when snails were not present (Contrast 3: *t*_20_ = −2.60; *p* = 0.02; Figure 1d). In the absence of snails, free‐swimming water bugs only stimulated tadpole growth by 20% and this effect was not statistically different from zero (Contrast 5b: *t*_20_ = 1.66; *p* = 0.11). Caged water bugs had little effect on tadpole growth either in the presence (Contrast 4b: *t*_20_ = 1.37; *p* = 0.19) or absence (Contrast 4c: *t*_20_ = 1.04; *p* = 0.31) of snails and these effects did not statistically differ from each other (Contrast 2: *t*_20_ = 1.70; *p* = 0.10; Figure 1d). Differences in the effect of free‐swimming and caged water bugs on tadpole growth did not depend on whether tadpoles cooccurred with snails (Contrast 6: *t*_20_ = −0.89; *p* = 0.39) but, on average, tadpoles grew 41% faster with free‐swimming water bugs than with caged water bugs (Contrast 7a: *t*_20_ = −5.17; *p* < 0.001; Figure 1d).

**Figure 3:**
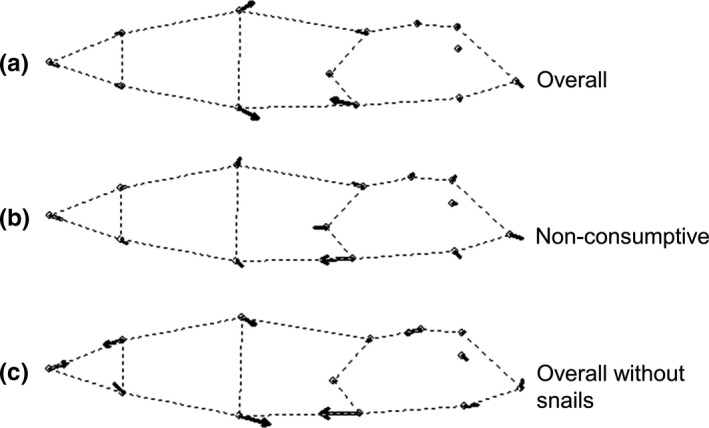
Landmark vectors illustrating tadpole shape change in response to: (a) free‐swimming water bugs (representing the overall effect of water bugs) with snails relative to tadpole shape in the absence of water bugs with snails (Contrast 5c), (b) nonconsumptive water bugs with snails relative to those in the absence of water bugs with snails (Contrast 7c), and (c) free‐swimming water bugs (overall effect) without snails relative to nonconsumptive water bugs without snails (Contrast 7b). Vector length represents the magnitude and direction of shape change for the associated landmark

The effect of free‐swimming predators on tadpole shape was more apparent when tadpoles cooccurred with snails (Contrast 5c: *F*_6, 9_ = 4.97; *p* = 0.02; $\eta_{p}^{2}$ = 0.77) than when they did not (Contrast 5b: *F*_6, 9_ = 1.23; *p* = 0.37; $\eta_{p}^{2}$ = 0.45). Nonetheless, we only have weak statistical evidence to reject the hypothesis that the effects were the same in each environment (Contrast 3: *F*_6, 9_ = 2.23; *p* = 0.13; $\eta_{p}^{2}$ = 0.60). In general, free‐swimming water bugs caused tadpoles to alter their shape (Contrast 5a: *F*_6, 9_ = 6.61; *p* = 0.006; $\eta_{p}^{2}$ = 0.82). Free‐swimming water bugs produced tadpoles with greater tail depth and larger head size (Figure 3a).

Caged water bugs had no effect on tadpole shape (Contrast 4a: *F*_6, 9_ = 0.93; *p* = 0.52; $\eta_{p}^{2}$ = 0.38) and this effect did not depend on whether snails were present (Contrast 2: *F*_6, 9_ = 1.15; *p* = 0.41; $\eta_{p}^{2}$ = 0.43). Consequently, free‐swimming water bugs and caged water bugs differed in their effect on tadpole shape (Contrast 7a: *F*_6, 9_ = 6.38; *p* = 0.007; $\eta_{p}^{2}$ = 0.81), but the specific way in which they differed depended on whether snails were present (Contrast 6: *F*_6, 9_ = 3.26; *p* = 0.05; $\eta_{p}^{2}$ = 0.69). Free‐swimming water bugs and caged water bugs differed in their effects on tadpole shape when snails were absent (Contrast 7b: *F*_6, 9_ = 5.99; *p* = 0.009; $\eta_{p}^{2}$ = 0.80). Tadpoles had larger heads in the presence of free‐swimming water bugs (Figure 3b). Free‐swimming water bugs and caged water bugs also differed in their effects on tadpole shape when snails were present (Contrast 7c: *F*_6, 9_ = 5.54; *p* = 0.01; $\eta_{p}^{2}$ = 0.79), but in this case tadpoles had both larger heads and deeper tails in the presence of free‐swimming water bugs (Figure 3c).

**Figure 4:**
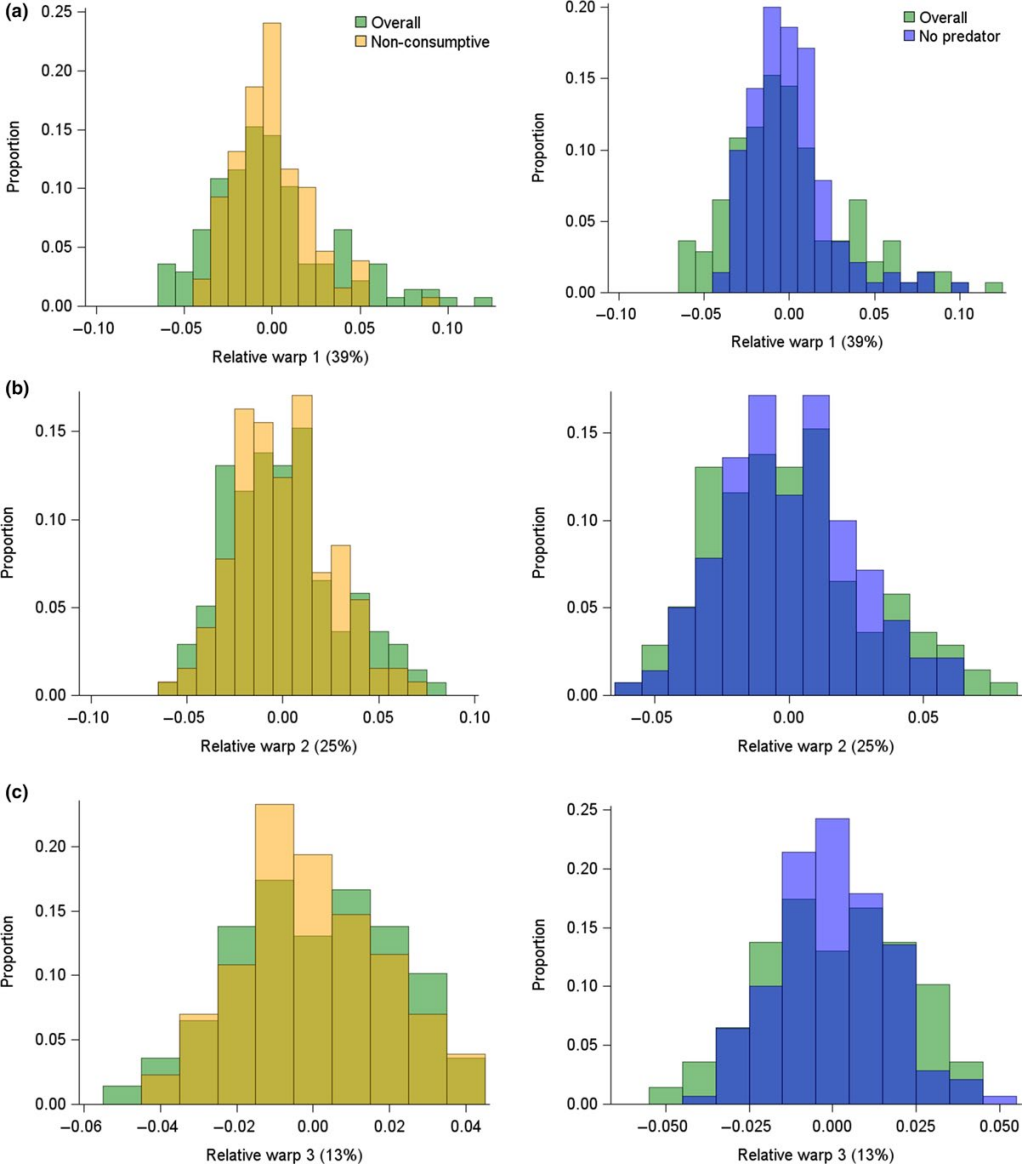
Distribution of standardized tadpole shape scores within mesocosms for the first three principal components (i.e., relative warps) of shape (a–c). On the left are distributions for treatments with (i) free‐swimming water bugs (overall predator effect, green) and (ii) caged water bugs (nonconsumptive predator effect, orange). On the right are distributions for treatments with (i) free‐swimming water bugs (overall predator effect, green) and (ii) no water bugs (blue). The top, a, middle, b, and bottom, c, panels refer to three different but independent measures of tadpole shape that together explain 77% of the overall variation in tadpole shape as described in the methods [Colour figure can be viewed at http://wileyonlinelibrary.com]

The shape of the distribution of standardized tadpole shape scores within mesocosms did not differ substantially between the no‐predator and caged‐predator treatments regardless of the shape variable (all *p* ≥ 0.499). We did observe that the distribution of standardized prey shape scores for mesocosms with free‐swimming predators differed from those observed in mesocosms with either no predators or caged predators for the first shape variable (*p* ≤ 0.057, Figure 4a), but not other shape variables (all *p* ≥ 0.217, Figure 4b, c). The distribution of scores for the first standardized shape variable was broader in the treatment with free‐swimming predators than in the other treatments (Figure 4a). If predators were selectively removing individuals on one end of the phenotypic distribution of standardized shape scores the distribution would appear truncated with fewer standardized shape scores on that end of the distribution, but we did not observe this.

### 3.4 Effects of prey on periphyton

**Figure 5:**
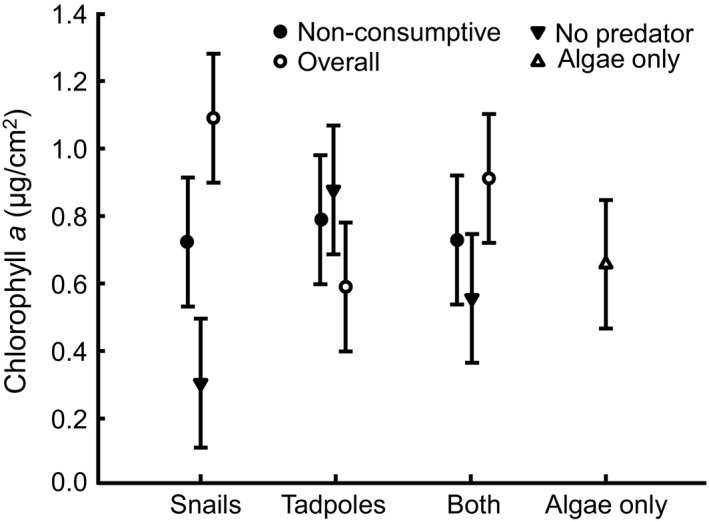
Chlorophyll *a* density (mean ± *SE*) of periphyton growing on plastic flagging tape in mesocosms in response to nonconsumptive water bugs (nonconsumptive effect, filled circle), free‐swimming water bugs (overall effect, open circle), no water bugs (filled triangle), and no water bugs or prey as a reference (open triangle). Snails and tadpoles occurred together at half the density of mesocosms with a single prey species

Tadpoles and snails differed in their ability to suppress algae (Contrast 8: *t*_40_ = 2.12; *p* = 0.04; Figure 5). Relative to treatments with no grazers, snails reduced algal density by 54% while tadpoles enhanced algal density by 34%, but neither of these effects were statistically different from zero (effect of snails ‐Contrast 9b: *t*_40_ = 1.31; *p* = 0.20; effect of tadpoles ‐Contrast 9c: *t*_40_ = 0.82; *p* = 0.42; Figure 5). Algal density did not depend on whether mesocosms contained one or two prey species (Contrast 10a: *t*_40_ = 0.15; *p* = 0.88).

### 3.5 Indirect effects of predators on periphyton

The effect of free‐swimming water bugs on algal density depended on which prey species was present (Contrast 12: *t*_40_ = 2.81; *p* = 0.008; Figure 5), but not on how many prey species were present (Contrast 16a: *t*_40_ = 0.32; *p* = 0.75). Free‐swimming water bugs enhanced algal density by 264% when snails were present but reduced algal density by 33% when tadpoles were present; only the effect of free‐swimming water bugs in the presence of snails was statistically different from zero (presence of snails ‐contrast 14c: *t*_40_ = 2.91; *p* = 0.006; presence of tadpoles ‐contrast 14b: *t*_40_ = 1.07; *p* = 0.29; Figure 5).

Caged predators caused algal abundance to increase by 140% when snails were present (Figure 5). Although this effect was not statistically different from zero (Contrast 13c: *t*_40_ = 1.55; *p* = 0.13), it was also not statistically different from the effect of free‐swimming water bugs on algae when snails were present (Contrast 17a: *t*_40_ = −1.36; *p* = 0.18). Any other effects of caged predators on algal abundance were weak and not statistically different from zero (|*t*| ≤ 1.33; *p* ≥ 0.19) or were not different from the effect of free‐swimming predators (|*t*| ≤ 1.68; *p* ≥ 0.10).

## 4 DISCUSSION

The primary goal of our study was to evaluate how differences in the relative susceptibility of prey to interspecific and intraspecific competition causes the nonconsumptive and overall effect of predators on their prey to change depending on whether multiple prey species cooccur. In our study system, snails were more vulnerable to intraspecific than interspecific competition. Snails grew 54% faster when some of the snails were replaced with tadpoles even though the total initial number of competitors (tadpoles + snails) was the same. Though the presence of snails did not cause a statistically significant change in tadpole response variables when predators were absent, other evidence suggests that tadpoles were more susceptible to interspecific competition. This evidence includes (1) Tadpole mortality risk more than doubled in the absence of predators when tadpoles and snails cooccurred than when snails were absent and (2) overall, snails and tadpoles differed in their ability to suppress algal abundance. Snails reduced algal abundance, while tadpoles enhanced algal abundance.

### 4.1 Predator effects on snails

Predators exerted strong effects on the mortality risk and growth of snails. Given that snails were more susceptible to intraspecific than interspecific competition, we expected that the presence of tadpoles would strengthen the effect of predators on snail growth but weaken their effect on snail mortality. Water bugs were very effective snail consumers, but their effect on snail mortality risk varied little between environments differing in the presence of tadpoles. In contrast, our observed effects of predators on snail growth appeared consistent with predictions. We found more evidence to suggest that predators exerted an effect on snail growth when tadpoles and snails cooccurred compared to when they did not cooccur. Furthermore, the magnitude of the estimated overall effect of predators on snail growth was greater when tadpoles and snails cooccurred (35% reduction in snail growth) relative to when they did not cooccur (4% reduction in snail growth).

In the presence of tadpoles, the predator‐induced reduction in snail growth that we observed is consistent with what we would expect as the result of reduced foraging activity by snails (a NCE) (Peacor & Werner, 2004; Ruehl & Trexler, 2013; Turner, Fetterolf, & Bernot, 1999). Consumptive mechanisms that reduce snail abundance often enhance body growth of surviving snails (e.g., Turner, 2004), and we did not observe this outcome. Furthermore, if predators caused a change in mean snail growth as a result of killing either faster or slower growing snails (i.e., a consumptive mechanism distinct from changing prey abundance) we would have observed many fewer individuals on one side of the standardized growth score distribution in mesocosms with uncaged predators than we observed without uncaged predators. However, there was no variation in growth score distributions (Figure 2). We were surprised that caged predators (which could only affect prey via nonconsumptive mechanisms) did not cause a change in snail body growth in our study and provide a possible explanation for the absence of caged predator effects below.

We believe that the perceived weaker overall effect of predators on snail growth when tadpoles were absent was not due to a weakening of nonconsumptive mechanisms through which predators affected snails. If nonconsumptive mechanisms affected snail growth less when tadpoles were absent we should have observed snails growing faster in environments with predators when tadpoles were absent than when tadpoles were present. We did not observe this—snails grew at a similarly slow rate in the presence of predators regardless of whether tadpoles were present. Furthermore, we would expect snails to grow more quickly with predators than without predators in environments lacking tadpoles because predators caused an increase in the amount of algae present (by killing or reducing the foraging activity of snails; Figure 5) which should have facilitated snail body growth (Ruehl & Trexler, 2013; Turner, 2004). We did not observe snails taking advantage of this potential growth opportunity. The most likely explanation for why snails did not take advantage of this growth opportunity is that the fear of predation altered their foraging behavior, conversion efficiency, metabolism, or some other nonconsumptive means. Consequently, nonconsumptive mechanisms appear to contribute to the slow growth of snails in the presence of predators regardless of whether tadpoles were present.

If predators exert an important effect on snail growth regardless of the presence of tadpoles, what caused the overall effect of predators on snail growth to weaken when tadpoles were absent? We believe the best explanation for why the overall effect of predators (i.e., difference in snail growth between environments having or lacking predators) weakened in the absence of tadpoles compared to when tadpoles were present is that there was a change in the importance of mechanisms operating in the absence of predators. Specifically, the intensity of competitive interactions operating on snails is stronger when tadpoles have been replaced with snails so snails grow more slowly in the absence of tadpoles (and predators) than in the presence of tadpoles (but no predators). In fact, the replacement of tadpoles with snails in environments lacking predators was sufficient to produce the same suppressive impact on snail body growth that nonconsumptive mechanisms from predators because snail body growth did not appear to differ much among environments except when tadpoles were present and predators were absent (Figure 1b).

### 4.2 Predator effects on tadpoles

Predators exerted strong effects on tadpole morphology, growth and mortality risk. We expected that the cooccurrence of snails with tadpoles would (1) not alter the effect of predators on tadpoles if we assume that tadpoles are equally susceptible to intraspecific and interspecific competition or (2) weaken the effect of predators on tadpole growth and development but strengthen the effect of predators on tadpole mortality risk if we assume that tadpoles are more susceptible to interspecific competition. We found that tadpole mortality risk to predators differed little between environments that varied in whether snails and tadpoles cooccurred, which was consistent with our conclusion that tadpoles were equally susceptible to intraspecific and interspecific competition. However, predator effects on tadpole morphology and growth were not consistent with either expectation as (1) we have stronger evidence to indicate that predators exerted an effect on tadpole growth and morphology when snails occurred than when they did not occur and (2) the magnitude of the effect of predators on tadpole morphology and growth was stronger when snails occurred with tadpoles (effect sizes: 55% increase in tadpole growth and 77% of variance in tadpole morphology explained) than when they did not (effect sizes: 20% increase in tadpole growth and 45% of variance in tadpole morphology explained).

We believe that predators largely affected tadpole growth via consumptive mechanisms and that consumptive mechanisms were stronger when tadpoles and snails cooccurred. Evidence in support of the idea that consumptive mechanisms primarily explain the impact of predators on tadpole growth is that tadpoles increased their growth rate in response to the presence of predators (Figure 1d). The increase in tadpole growth can be attributed to uncaged predators alleviating the effects of competition on individual tadpoles by eating their intraspecific and interspecific competitors. In contrast, a nonconsumptive mechanism through which predators can affect body growth of prey is by inducing prey to lower their foraging activity, as lowered foraging activity reduces prey growth (Ruehl & Trexler, 2013; Turner, 2004; Werner & Peacor, 2003). Tadpoles did not grow more slowly in the presence of predators so we do not believe that predators exerted an important NCE on tadpole growth directly. As we note above, however, predators likely caused snails to reduce their foraging activity which would free up food resources. Consequently, predators could have indirectly facilitated tadpole growth by reducing snail foraging activity as suggested by Peacor and Werner (2004). We do not believe, however, that this indirect influence of a NCE of predators exerted as strong of an effect on tadpole growth as the consumptive mechanism. Though predators caused a 42% reduction in snail foraging activity when tadpoles were present (assuming foraging activity is proportional to snail growth as per Peacor and Werner (2004)), predators enhanced mortality risk of snails by 193% and tadpoles by 24% (Figure 1a,c vs. Figure 1d). Consequently, the killing of large numbers of competitors likely had a greater influence on food availability for tadpoles than smaller proportional reductions in snail foraging activity.

In contrast to the effect of predators on tadpole growth, we believe that predators affected tadpole morphology via nonconsumptive mechanisms. Consumptive mechanisms would have altered tadpole shape by removing individuals with phenotypes that made them susceptible to predation and we would have observed many fewer individuals on one side of the standardized shape score distribution in mesocosms with uncaged predators than we observed without uncaged predators. We did not observe this (Figure 4a–c). Furthermore, the change in tadpole morphology that we observed was consistent with what we would expect on the basis of prior knowledge about how nonconsumptive mechanisms cause predators to affect tadpole morphology. Predators caused tadpoles, when they cooccurred with snails in our study, to exhibit a body shape that was characteristic of tadpoles raised in the presence of nonconsumptive (e.g., caged) predators (Relyea, 2001, 2002; Werner & Peacor, 2006). We were surprised that caged predators (which could only affect prey via nonconsumptive mechanisms) did not cause a change in tadpole shape in our study, but we provide an explanation for this below.

We expected that stronger interspecific competition with snails would reduce the per capita availability of food resources for tadpoles and thereby weaken the NCE of predators on tadpole shape. Instead the NCE of predators on tadpole shape was stronger in the presence of snails. We believe per capita food availability for tadpoles actually increased in the presence of snails and predators because snails were more vulnerable to predation than tadpoles and the greater loss of superior competitors (snails) resulted in greater per capita availability of food for surviving tadpoles. Algal abundance did not vary predictably among treatments with tadpoles (Figure 5) which supports the notion that there would be a greater per capita food availability for surviving tadpoles in environments that experienced higher mortality of superior grazers. The greater per capita availability of food resources for tadpoles in environments containing both snails and predators also explains why predators enhance tadpole growth when snails are present (see above). Consequently, our observations suggest that stronger interspecific competitors can enhance the NCE of predators on prey morphology when the interspecific competitor is more vulnerable to predation but weaken the NCE of predators when they are less vulnerable to predation.

### 4.3 Why did caged predators exert weak effects when nonconsumptive mechanisms best explain the effects of uncaged predators?

We expected that the NCE of predators would manifest themselves in treatments with caged predators because predators could not capture and consume prey in this treatment, but we did not observe these effects. An insufficient supply of water‐borne predator cues emanating from predator cages might explain why caged predators generally exerted weak effects on prey. However, we believe sufficient cue was provided because (1) we have previously documented NCE stemming from predators placed into the same type of cage used in this study (Davenport & Chalcraft, 2013) and (2) the concentration of prey that we fed to predators on a daily basis (1.14 prey items/day) was similar to or exceeded that fed to predators in other studies that report NCEs (Range: 0.85–2.14, Van Buskirk & Relyea, 1998; Peacor & Werner, 2001; Turner, 2004; Wojdak & Luttbeg, 2005; Werner & Peacor, 2006; Hoverman & Relyea, 2007, 2012; Davenport & Chalcraft, 2013; Ruehl & Trexler, 2013). Another possibility affecting the amount of water‐borne chemical cue available to prey species is that mixed species treatments received less cue than single species treatments. However, if this had been an important effect, single species treatments would have responded differently than mixed species treatments and we did not find evidence that the single species and mixed species treatments responded differently in mesocosms with caged predators.

Caged predators might have exerted weak effects on prey if stimuli, in addition to chemical cues, were needed to trigger nonconsumptive responses of prey to predators. If one form of stimuli could not adequately characterize the risk to prey, multiple sensory modes that provided information on the level of threat may be required to elicit a response by prey (Munoz & Blumstein, 2012; Sih, 1986). For example, chemical signals could alert prey to the presence of predators, but prey may never encounter predators. Therefore, prey may need to have direct encounters with predators, in addition to detecting chemicals that signal the presence of predators, before inducing costly anti‐predator traits (e.g., morphological changes) that may reduce fitness. The need for prey to receive multiple stimuli before producing a NCE of predators could pose a substantial challenge for studies quantifying NCEs using methods like caging that reduce the number and/or kinds of stimuli available to prey. We suggest including free‐swimming predator treatments in addition to caged treatments to determine the limitations of caging treatments and quantify the overall effects of predators on the system. Nonetheless, there is ample evidence to indicate that multiple stimuli are not always necessary to provoke NCEs given that some prey species do respond to the presence of caged predators (Davenport & Chalcraft, 2013; Ruehl & Trexler, 2013; Schmitz, Hamback, & Beckerman, 2000).

### 4.4 Cascading effects of predators on algae

We expected that any consumptive or NCEs of predators on their prey would have cascading impacts on the abundance of food available to prey. Even though predators exerted strong impacts on both their snail and tadpole prey, we observed little evidence that these effects translated into important effects on algae. Predators effects in the presence of snails were the only exception to this trend. Algal abundance increased tremendously in response to free‐swimming water bugs (relative to when water bugs were absent) when snails were the only prey present. Such a strong trophic cascade likely occurred because snails were more efficient at harvesting algal resources and were more vulnerable to predation compared to tadpoles. Any impact of free‐swimming predators on algal abundance mediated through snails when they cooccurred with tadpoles would be minimized because of the much lower initial density of snails in those treatments. Though the effect of caged predators on algal abundance when snails were the only prey present was not statistically different from zero, the observed effect of caged predators on algal abundance was rather large and not statistically different from that effect observed with free‐swimming predators. This suggests that NCEs accounted for some of the overall effect of water bugs on algal abundance when snails were the only prey present. Consequently, predators may be more likely to induce trophic cascades when the prey present are (i) in high abundance, (ii) effective at suppressing primary producers, and (iii) vulnerable to both the consumptive and NCEs of predators.

## 5 CONCLUSIONS

Our results support the idea that predators can affect their prey via both consumptive and nonconsumptive mechanisms and that the overall impact of predators on a particular prey species depends on whether an inferior or superior interspecific competitor is present. We expected predator effects on prey to change when other prey species were present because differences in the susceptibility of a prey species to interspecific and intraspecific competitors would alter the strength of nonconsumptive mechanisms through which predators interact with their prey. Our findings support the expectation that the overall effect of predators on prey was due to a change in the strength of nonconsumptive mechanisms in one case (tadpole morphology) but not in two other cases (tadpole and snail body growth).

In one case (snail body growth), the overall effect of a predator on the body growth of a competitively superior prey species (snails) changed because of how prey responded to a change in the intensity of competition rather than a change in the strength of mechanisms through which predators affected prey. The superior competitor took greater advantage of the available resources when some of them were replaced with an inferior competitor, but only in the absence of predators. The threat of predation caused competitively superior prey to perform the same way regardless of whether competitively inferior prey was present or not. In a second case (tadpole body growth), predators primarily exerted a stronger positive effect on the body growth of the inferior competitor when the superior competitor was present because predators killed lots of the superior competitor that freed up food resources for surviving competitively inferior prey to grow. This is the primary mechanism through which some prey species benefit from a keystone predator (Paine, 1966). In the third case (tadpole morphology), inferior competitors responded to the threat of predation better when the superior competitor was present. Superior competitors were more vulnerable to predators (i.e., a consumptive mechanism) leaving more resources for the competitively inferior prey to invest in defenses (i.e., altered morphology) in response to the threat of predation (i.e., a nonconsumptive mechanism). Interestingly, in this case the strength of the nonconsumptive mechanism depended on the ability of the consumptive mechanism to suppress competition. Both the second and third cases require a trade‐off between the ability of a prey species to compete and their vulnerability to predation, but only the third case involves a change in the strength of the nonconsumptive mechanism through which predator and prey interact.

Nonconsumptive mechanisms played some role in the effect of predators on each prey species but the body growth of snails was more sensitive to nonconsumptive mechanisms from predators than tadpole body growth. One possible explanation for this discrepancy between prey species is that individuals of one prey species (tadpoles) must grow sufficiently to complete metamorphosis before a pond dries while the other prey species (snails) may have an opportunity to reproduce after a pond refills from drying. Tadpoles cannot reproduce themselves and will die when a pond dries. Conversely, pulmonate snails in general and physids in particular can reach sexual maturity rapidly under warm temperatures (Crowl & Covich, 1990; Dillon, 2000), much faster than tadpoles, can reproduce at least once within a single hydrological cycle of an ephemeral pond (Caquet, 1993; DeWitt, 1955), and can burrow into pond bottoms to wait until the pond refills (Alyakrinskaya, 2004; Boss, 1974; Fretter & Peake, 1979). Consequently, tadpoles may be less likely to alter their foraging behaviors to a degree that adversely affects their body growth than would snails (e.g., Peacor & Werner, 2000).

Our findings suggest at least two fruitful areas for future study. First, studies should explore how differences in the vulnerability of prey to predators and their susceptibility to intraspecific and interspecific competition affects the consumptive and nonconsumptive mechanisms through which predators affect their prey. Our work indicates that the mechanisms through which predators affect prey in different communities can be varied and complex. Second, integrating hypotheses and methods from work on multisensory responses in organisms (e.g., Higham & Hebets, 2013) might provide greater insight into the variable nonconsumptive responses of prey species to predators and this can often be done by including free‐swimming predator treatments into experimental designs that simultaneously measure the overall effect of predators.

## CONFLICT OF INTEREST

None declared.

## AUTHOR CONTRIBUTIONS

CBR, DRC, and HVC conceived and designed the experiments. CBR performed the experiments. CBR and DRC analyzed the data and wrote the manuscript. HVC helped with field work and provided editorial advice.

## DATA ACCESSIBILITY

Data will be made available through the Dryad Digital Repository.
