# Supplementary material for: Cooccurrence of prey species alters the impact of predators on prey performance through multiple mechanisms
Source: Ecol Evol. 2018 Aug 11;8(17):8894–907. doi: 10.1002/ece3.4413 (PMC6157665; doi:10.1002/ece3.4413)
Supplement: Supplementary file 1 [file ECE3-8-8894-s001.docx]

**Supplementary material Appendix 1 - Detailed methods and results**

*Collection of prey and their assignment to mesocosms:* Tadpoles used as prey for the experiment were newly hatched larvae from eight pairs of adult *H. chrysoscelis* collected while in amplexus at a fishless pond on the night of 16 June 2010. Snails used as prey were the offspring of 108 adult *P. acuta* that we placed in four 1100-l mesocosms on 20 April 2010, two months prior to the start of the experiment. We counted 50 newly hatched tadpoles into each of 45 containers. Siblings were evenly distributed among all containers. Juvenile snails were haphazardly counted into containers in lots of 100 or 50 snails. Tadpoles (mean mass±1SE, 5.85±0.11mg, estimated from three groups of 50 tadpoles) and snails (mean individual mass±1 SE, 4.77±0.11mg, n=300 from subsample of each mesocosm) were added to mesocosms on 23 June 2010. Mesocosms designated to contain only snails received one randomly selected container of 100 snails while mesocosms designated to contain snails and tadpoles received one randomly selected container with 50 snails. Mesocosms designated to contain only tadpoles received two randomly selected containers of 50 tadpoles while mesocosms designated to contain snails and tadpoles received one randomly selected container of 50 tadpoles.

*Estimation of prey mortality rates (m)*: Given that no snails survived in five of 10 replicates containing free-swimming water bugs, we added a one to the numerator of the proportion of individuals that survived in all mesocosms. Though this may lead to a slight underestimate of mortality rates, this addition was necessary to obtain a mortality estimate for mesocosms with no survivors. We do not think this addition impacted our results, however, because our interpretation of an analysis on m (described below) was qualitatively similar to the interpretation of an analysis on survival data (proportion surviving) with a generalized linear mixed model that used a quasi-likelihood approach that modeled a variance function since the residuals from an analysis implementing a binomial distribution were underdispersed.

*Estimation of body growth:* Body growth of the average prey individual in a mesocosm was estimated as ((average mass of individuals at the end of the experiment – average mass of individuals at the start of the experiment)/duration of the experiment). The body mass of each surviving animal was measured at the end of the experiment but we did not measure the body mass of every individual at the start of the experiment to avoid mortalities due to handling. Consequently, we estimated the body mass of the average animal at the start of the experiment. For tadpoles, we did this by 1) weighing three replicates of 50 tadpoles (in bulk) at the start of the experiment, 2) dividing by 50 to estimate individual mass, and 3) averaging the three individual mass estimates. For snails, we did this by 1) determining the shell length of 10 snails in each container of snails that were assigned to mesocosms and 2) estimating their individual biomass by applying a species specific equation that relates shell length to snail soft tissue biomass (Turner, unpublished model), and 3) averaging the individual estimates of soft tissue biomass. In cases where no individuals survived in a mesocosm (five of 10 replicates for snails with free-roaming water bugs) we could not derive an estimate of individual body growth.

*Geometric morphometric analysis to characterize body shape:* Using tpsDIG ([Rohlf 2008](#_ENREF_3)) software, we digitized 13 homologous landmarks of the tadpole lateral view and 7 homologous landmarks plus 2 semi-landmarks of the snail shell as it rested aperture side down (sensu: [Dayton, Saenz, Baum, Langerhans and DeWitt 2005 for tadpoles](#_ENREF_1), [Langerhans and DeWitt 2002 for snails](#_ENREF_2)). This resulted in 26 and 18 landmark coordinates (x + y) for tadpoles and snails respectively that were adjusted for position, scale, and orientation with a generalized Procrustes superimposition using tpsRELW ([Rohlf 2008](#_ENREF_4)) software. We conducted a principal components analysis of the covariance matrix of the superimposed landmark data. The first six principal components describing tadpole shape (i.e., six shape variables) accounted for 90% of overall shape variation and the first five principal components describing shell shape accounted for 91% of overall shape variation. These components served as our descriptors of animal shape in statistical analyses testing for variation in shape among treatments.

*Estimation of algal abundance:* Periphytometers were thawed and periphyton was scraped into a Petri dish, washed into a 10-ml graduated cylinder with DI water, diluted to a known volume with DI water, filtered onto a 2.5 mm glass-fiber filter, wrapped in foil, and then frozen. The total area of each periphytometer that was scraped was recorded to estimate chlorophyll *a* density. Chlorophyll *a* was extracted with acetone and read 24 hours later on a Milton Roy Spectronic 1201spectrophotometer at 665 nm before and after addition of 6N HCL to determine phaeophytin concentration and chlorophyll *a* density (μg/cm^2^).

**References**

Dayton, G. H., et al. 2005. Body shape, burst speed and escape behavior of larval anurans. - Oikos 111: 582-591.

Langerhans, R. B. and DeWitt, T. J. 2002. Plasticity constrained: Over-generalized induction cues cause maladaptive phenotypes. - Evol. Ecol. Res. 4: 857-870.

Rohlf, F. J. 2008. tpsDig. - Department of Ecology and Evolution, State University of New York.

Rohlf, F. J. 2008. tpsRelw. - Department of Ecology and Evolution, State University of New York.

**Supplementary material Appendix 2.** Statistical contrasts to evaluate effects of predators and competitors on a particular prey species. A verbal description of the effect being quantified is reported in the Description column. Letters following the number in the Contrast column differentiate hypotheses that are variations on a very similar question. For example, Contrast 4 generally assessed the NLE of predators but was assessed under the scenarios when one or two prey species were present (4a), one prey species was present (4b) or two prey species were present (4c). Treatment columns are differentiated on the basis of 1) whether caged predators, uncaged predators, or no predators were present and 2) whether snails (S), tadpoles (T), or both were present. Numbers in the treatment columns refer to contrast coefficients.

|  |  | Cage | Cage | Overall | Overall | No predator | No predator |
| --- | --- | --- | --- | --- | --- | --- | --- |
| Contrast | Description | Both | S or T | Both | S or T | Both | S or T |
| 1 | Is the species affected more by interspecific or intraspecific competition? | 0 | 0 | 0 | 0 | 1 | -1 |
| 2 | Does the NCE of predators depend on whether a species occurs alone versus with another prey species? | -1 | 1 | 0 | 0 | 1 | -1 |
| 3 | Does the overall effect of predators depend on whether species occur alone versus with other prey species? | 0 | 0 | 1 | -1 | -1 | 1 |
| 4a | What is the NCE of predators on prey? | -1 | -1 | 0 | 0 | 1 | 1 |
|  | If 4a is important estimate 4b and 4c instead. |  |  |  |  |  |  |
| 4b | What is the NCE of predators on prey when prey occurs alone? | 0 | -1 | 0 | 0 | 0 | 1 |
| 4c | What is the NCE of predators on prey when prey occurs with other prey species? | -1 | 0 | 0 | 0 | 1 | 0 |
| 5a | What is the overall effect of predators on prey? | 0 | 0 | -1 | -1 | 1 | 1 |
|  | If 5a is important estimate 5b and 5c instead. |  |  |  |  |  |  |
| 5b | What is the overall effect of predators on prey when prey occurs alone? | 0 | 0 | 0 | -1 | 0 | 1 |
| 5c | What is the overall effect of predators on prey when prey occurs with other prey species? | 0 | 0 | -1 | 0 | 1 | 0 |
| 6 | Does the difference between the overall and NCE differ depend on whether prey co-occur? | 1 | -1 | -1 | 1 | 0 | 0 |
| 7a | Do the NCE differ from the overall effect? | 1 | 1 | -1 | -1 | 0 | 0 |
|  | If 7a is important estimate 7b and 7c instead. |  |  |  |  |  |  |
| 7b | Does the NCE differ from the overall effect when prey occurs alone? | 0 | 1 | 0 | -1 | 0 | 0 |
| 7c | Does the NCE differ from the overall effect when the prey co-occurs with other prey? | 1 | 0 | -1 | 0 | 0 | 0 |

**Supplementary material Appendix 3.** Statistical contrasts to evaluate effects of predators and prey on algal abundance. A description of the effect being quantified appears in the description column. Letters following the numbers in the Contrast column differentiate hypotheses that are variations on a similar question. For example, Contrast 8 generally assesses the NCE of predators but was assessed when one or two prey species were present (10a), when tadpoles were the only prey species present (10b) or when snails were the only prey species present (10c). Treatment columns are differentiated on the basis of 1) whether caged predators, uncaged predators, or no predators were present and 2) whether snails, tadpoles, both, or no prey were present. Numbers in the treatment columns refer to contrast coefficients.

|  |  | Cage | Cage | Cage | Lethal | Overall | Overall | No Predator | No Predator | No Predator | No Predator |
| --- | --- | --- | --- | --- | --- | --- | --- | --- | --- | --- | --- |
| Contrast | Description | Both | Snail | Tadpole | Both | Snail | Tadpole | Both | No Prey | Snail | Tadpole |
| 8 | Do grazer species differ in their ability to suppress algae? | 0 | 0 | 0 | 0 | 0 | 0 | 0 | 0 | 1 | -1 |
| 9a | What is effect of a single grazer species on algae? | 0 | 0 | 0 | 0 | 0 | 0 | 0 | 2 | -1 | -1 |
| 9b | What is the effect of snails on algae? | 0 | 0 | 0 | 0 | 0 | 0 | 0 | 1 | -1 | 0 |
| 9c | What is the effect of tadpoles on algae? | 0 | 0 | 0 | 0 | 0 | 0 | 0 | 1 | 0 | -1 |
| 10a | Does number of grazer species affect algae? | 0 | 0 | 0 | 0 | 0 | 0 | -2 | 0 | 1 | 1 |
| 10b | What is the effect of both grazers versus snails on algae? | 0 | 0 | 0 | 0 | 0 | 0 | 1 | 0 | -1 | 0 |
| 10c | What is the effect of both grazers versus tadpoles? | 0 | 0 | 0 | 0 | 0 | 0 | 1 | 0 | 0 | -1 |
| 11 | Does the NLE depend on which species is present? | 0 | -0.5 | 0.5 | 0 | 0 | 0 | 0 | 0 | 0.5 | -0.5 |
| 12 | Does the overall effect depend on which species is present? | 0 | 0 | 0 | 0 | -0.5 | 0.5 | 0 | 0 | 0.5 | -0.5 |
| 13a | What is the NLE when one prey species is present | 0 | 0.5 | 0.5 | 0 | 0 | 0 | 0 | 0 | -0.5 | -0.5 |
| 13b | What is the NLE with tadpoles | 0 | 0 | 1 | 0 | 0 | 0 | 0 | 0 | 0 | -1 |
| 13c | What is the NLE with snails | 0 | 1 | 0 | 0 | 0 | 0 | 0 | 0 | -1 | 0 |
| 14a | What is overall effect when one prey species is present? | 0 | 0 | 0 | 0 | -0.5 | -0.5 | 0 | 0 | 0.5 | 0.5 |
| 14b | What is overall effect with tadpoles? | 0 | 0 | 0 | 0 | 0 | -1 | 0 | 0 | 0 | 1 |
| 14c | What is overall effect with snails? | 0 | 0 | 0 | 0 | -1 | 0 | 0 | 0 | 1 | 0 |
| 15a | Does the NLE depend on whether 1 prey species is present or 2? | 1 | -0.5 | -0.5 | 0 | 0 | 0 | -1 | 0 | 0.5 | 0.5 |
| 15b | Does the NLE depend on whether snail occurs alone? | 0.5 | -0.5 | 0 | 0 | 0 | 0 | -0.5 | 0 | 0.5 | 0 |
| 15c | Does the NLE depend on whether tadpoles occurs alone? | 0.5 | 0 | -0.5 | 0 | 0 | 0 | -0.5 | 0 | 0 | 0.5 |
| 16a | Does the overall effect depend on whether 1 prey species is present or 2? | 0 | 0 | 0 | 1 | -0.5 | -0.5 | -1 | 0 | 0.5 | 0.5 |
| 16b | Does the overall effect depend on whether snail occurs alone? | 0 | 0 | 0 | 0.5 | -0.5 | 0 | -0.5 | 0 | 0.5 | 0 |
| 16c | Does the overall effect depend on whether tadpoles occur alone? | 0 | 0 | 0 | 0.5 | 0 | -0.5 | -0.5 | 0 | 0 | 0.5 |
| 17a | Does the overall effect differ from NLE when snails present? | 0 | 1 | 0 | 0 | -1 | 0 | 0 | 0 | 0 | 0 |
| 17b | Does the overall effect differ from NLE when tadpoles present? | 0 | 0 | 1 | 0 | 0 | -1 | 0 | 0 | 0 | 0 |
| 17c | Does the overall effect differ from NLE when both present? | 1 | 0 | 0 | -1 | 0 | 0 | 0 | 0 | 0 | 0 |
| 17d | Does the overall effect differ from NLE when one versus both prey are present? | -1 | 0.5 | 0.5 | 1 | -0.5 | -0.5 | 0 | 0 | 0 | 0 |
